# Molecular Landscape and Computational Screening of the Natural inhibitors against HPV16 E6 Oncoprotein

**DOI:** 10.31557/APJCP.2021.22.8.2461

**Published:** 2021-08

**Authors:** Tanzil Juneja, Medha D Pandya, Sejal Shah

**Affiliations:** 1 *Department of Microbiology, School of Science, RK University, Rajkot, India. *; 2 *The KPES Science College, M.K Bhavnagar University, Bhavnagar, India. *

**Keywords:** Human Papillomavirus (HPV), E6 oncoprotein-, natural compounds, molecular docking

## Abstract

**Background:**

Human Papillomavirus (HPV) is a small, non-enveloped, icosahedral and double-stranded DNA virus with a genome of 8 kb, belonging to the papillomaviridae family. HPV has been associated with 99.7% cases of cervical squamous cell carcinoma worldwide. The HPV E6 protein is known as a potent oncogene and is closely allied with the events that result in the malignant transformation of virally infected cells.

**Objective:**

The present study aims to target plant derived anticancer molecules for HPV driven cancer using a computational approach. Methods: In this study, E6 oncoprotein was targeted by 101 plant-derived nutraceuticals using the molecular docking method. The multiple sequence analysis and phylogenetic analysis of low risk and high risk 28 HPV E6 proteins were performed.

**Results:**

Withanolide D, Ginkgetin, Theaflavin, Hesperidin, and Quercetin-3-gluconide were identified as the potential inhibitors of HPV 16 E6 protein. The zinc finger domain was identified on all variants of HPV E6 oncoprotein while high-risk HPV18, HPV31, HPV33, HPV35, HPV39, HPV45, HPV58, HPV68 and HPV73: probable risk HPV53 and low-risk HPV43 and HPV70 contain PDZ domain.

**Conclusion:**

The current study using bioinformatics analysis approaches reveals a promising platform for developing anti-cancerous competitive inhibitors targeting HPV.

## Introduction

**Figure 1:**
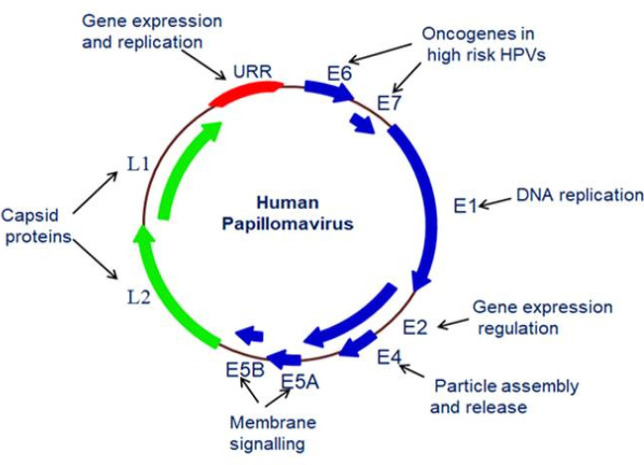
HPV Structure and Function of Viral Proteins

Cancer is a multifactorial disease, which involves the degradation of several cell signalling pathways (Shah et al., 2015; Shah, 2016; Shah et al., 2017; Shah et al., 2018) recent study identified various metabolites for onset of cancer (Patel et al., 2021). There are various risk factors responsible for developing cancer, including oncogenic viruses. Human papillomavirus (HPV) is a well-known oncogenic virus and the incidence of HPV-related cancer is higher across the world.. HPV has been associated with 90% of cervical squamous cell carcinoma cases worldwide, as well as some other cancers related to the vagina, vulva, penis, anus, head and neck, oral cavity and oropharynx (de Martel et al., 2020). Human papillomavirus (HPV) is a small, non-enveloped, icosahedral and double-stranded DNA virus with a genome of 8 kb, belonging to the Papillomaviridae family. More than 10% of human cancers are developed by the infection of HPV (Arbyn et al., 2020). On the basis of potential oncogenic HPVs can be categorized into two subsets: low-risk type and high-risk type (Table.1). HPV genome comprises three distinct regions: The early region, the late region, and URR (upstream regulatory region). The early part contains seven early proteins (E1, E2, E4, E5A, E5B, E6, and E7); the late includes two late proteins (L1 and L2); and promoter and enhancer region which is responsible for the gene regulation are covered in the URR part (Figure 1) (Pal and Kundu, 2020).

Consequently, HPV 16 and 18 are included in high-risk HPVs. Out of the eight types of proteins expressed in HPV, E6 and E7 proteins are reported as cooperative viral oncoprotein due to their expression in all HPV types. Naturally occurring chimeric proteins have a tendency to cause cancer hence, they are known as oncoprotein (Dave et al., 2015, Pandya et al., 2020, Daga et al., 2018). The HPV E6 protein is known as a potent oncogene and is closely allied with the events that result in the malignant transformation of virally infected cells (Mc Bride, 2017). The HPV E6 and the E7 proteins modulate cellular proteins that control the cell cycle. The E6 oncoprotein binds to the p53 tumour suppressor protein and targets it for accelerated ubiquitin-mediated degradation and telomerase activity (DeFilippis et al., 2003). E6 oncoprotein disturb transcriptional pathways, disrupt cell adhesion and architecture, inhibit apoptosis, abrogate DNA damage responses, induce genome instability and immortalize cells (Chan et al., 2002).

Based on their association with cervical cancer and precancerous lesions, HPVs can also be grouped into high-risk and low-risk HPV types. Low-risk HPVs are types: 6, 11, 40, 42, 43, 44, 54, 61, 70, 72, and 81. These types can cause genital lesions but are considered non-carcinogenic types as they are not associated with cancerous lesions and are very rarely associated with precancerous lesions. HPV 6 and 11 are the most common cause of genital condylomas (genital warts). High-risk HPVs are types: 16, 18, 31, 33, 35, 39, 45, 51, 52, 58, 59, 68, 73, and 82. These high-risk HPVs are associated with cervical as well as other anogenital cancers, and they are referred to as the carcinogenic or oncogenic HPV types. HPV 16 and 18 are the most common risk factors in cervical cancer; however, infections with HPV 16 or 18 do not always result in cancer (Harvey et al., 2015).

HPV is the well-known risk factor for the development of cervical cancer. Globally, 99.7% prevalence of HPV has been reported in cervical cancer. The rate of cervical cancer is higher in South-Eastern Asia with excessive burden especially in India, Latin America and Saharan Africa. (Turkhia et al., 2018). In Western asia specifically the prevalence of HPV infection in Iranian women is 29.3% (871/2969) where the frequency of high risk HPV infection is 584(19.7%) and low risk HPV infection is 453(15.3%) (Chalabiani et al., 2017).

Prevalence of HPV infection varies among different parts of India. Frequency of HPV infection is 51.3% in Eastern India, 15.5% in Western India, 48.6% in South India and 35.2% in North India (Patel et al., 2021) Especially in the western part of india, the frequency of the cervical cancer is high, 31 (59.6%) patients were infected with HPV 16 and HPV 18. Of these 31 HPV-positive cervical cancer patients, 28 (90.3%) were infected with HPV 16 and 3 (9.7%) were infected with HPV 18 (Patel et al., 2014). HPV 16 and HPV 18 positivity was observed in 56% and 15% cases, respectively, whereas 6% of the cases showed co-infection with both these types (Thobias et al., 2019).

Natural product is a chemical compound or substance produced by a living organism that is, found in nature. It has been a rich source for novels explored in drug discovery (Shah and Ghosh, 2020). These compounds show superior structural diversity, bioactivity and more complexity as compared to the compounds present in synthetic drug libraries. They have the capacity to constrain some targets which are considered “undruggable”, such as protein-protein interactions; all the natural products are secondary metabolites. Recent studies have clearly indicated the role of vitamins exhibits the antiviral property (Pandya et al., 2021). Natural products and synthetic drugs limit the overlapping. Drug repurposing study based on artificial nural network has shown higher affinity towards post viral prognosis (Menamadathil et al., 2021) These characteristics not only indicate the potential for new targets for therapy but also can help reduce the cost of the development of new treatments since these molecules already exist in nature, and these compounds offer additional options for combination therapies

In this study, we are focusing on a computational approach and target oncoprotein with plant-derived natural compounds. Computational approaches for drug discovery and development are valuable and significant tools. Several computational methodologies are appropriate in the identification and investigation of new drug candidates (Hung and Chen, 2014). We also performed the evolutionary analysis of different variants of E6 oncoprotein.

## Materials and Methods

*Protein and ligand preparation*

The high resolution three dimensional X-ray crystal structures of the E6 oncoprotein of HPV-16 retrieved from protein data bank (PDB) (http://www.rcsb.org/) using their accession IDs 2FK4. For plant-derived natural anticancer compounds, NPACT database is crucial; 101 natural compounds have been selected from this database (Table S1), which were further assembled into data-sets and used for the docking purpose. The protein and natural compounds were prepared for molecular docking using AutoDockTools 1.5.6. and OpenBabel 3.1.1 consequently.

*Molecular docking study*

The binding affinity of natural compounds with the E6 oncoprotein of HPV 16 targets was determined by molecular docking method. The molecular docking was performed using blind docking method in Autodock Vina 1.5.6 (Trott and Olson, 2010).The grid box is outsized enough to protein structure to encounter any probable protein-ligand interactions. The binding poses were clustered and ranked in the order of their binding affinities. The molecular interactions (hydrogen bonds and hydrophobic interactions) between the target proteins and compounds were studied using LigPlot + version 1.4.5 (Laskowski and Swidells, 2011).

*Multiple sequence alignment and phylogenetic analysis*

The multiple sequence analysis and phylogenetic analysis of low-risk and high-risk HPV E6 protein were performed. The protein sequence of 28 E6 oncoprotein was retrieved from the UniprotKB database (https://www.uniprot.org/) (Table 1). Multiple sequence alignment (MSA) of all sequences is performed using Clustal Omega (Chatzou et al., 2016). The phylogenetic analysis was performed in MEGA-X 10.2.0 (Kumar et al., 2018) and the tree was built using the neighbour joining method.

## Results

**Figure 2:**
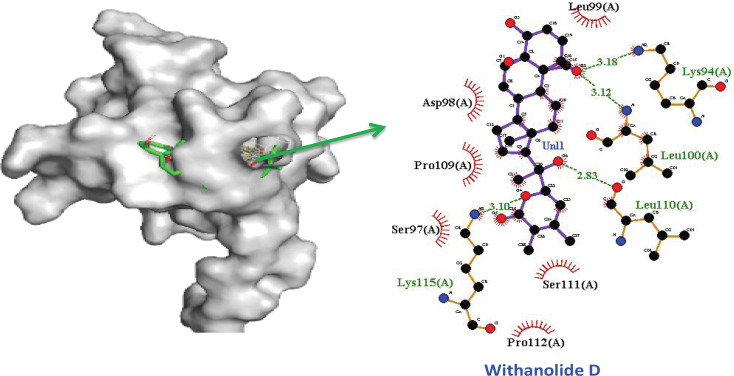
Protein Ligand Complex Representation for c-terminal Zinc Binding Domain of E6 Oncoprotein with Ligand Withanolide D

Currently, there are various conventional therapies for HPV infection, but they all of them have side-effects. More than 50% of all the drug forms used in the clinical fields around the world have originated from the compounds extracted from plants. Secondary metabolites of plants are known as splendid sources of medicinal compounds. Plants based compounds play a significant role with no side effects. In this bioinformatics study, the virtual screening of ligands against E6 oncoprotein achieved and Withnolide D, Ginkgetin, Theaflavin, Hesperidin, Quercetin-3-gluconide, Silymarin, Epigallocatechin gallate (EGCG), Flavonol 3-O-glycoside, Ginkgolides B and Curcumin were identified as potential inhibitors of HPV 16 E6 protein (See Table 2). Among all-natural compounds, Withnolide D demonstrated virtuous affinity -7.5 kcal/mol with E6 oncoprotein. The interacting residues of E6 oncoprotein with Withnolide D confirmed in Figure 2 with the context to hydrophobic contact and H-bond are Leu99,Ser111, Pro112, Ser97, Pro109, Asp98 and Lys94 (3.18Å), Leu100 (3.12Å), Leu110 (2.83Å) and Lys115 (3.10Å) .

**Figure 3:**
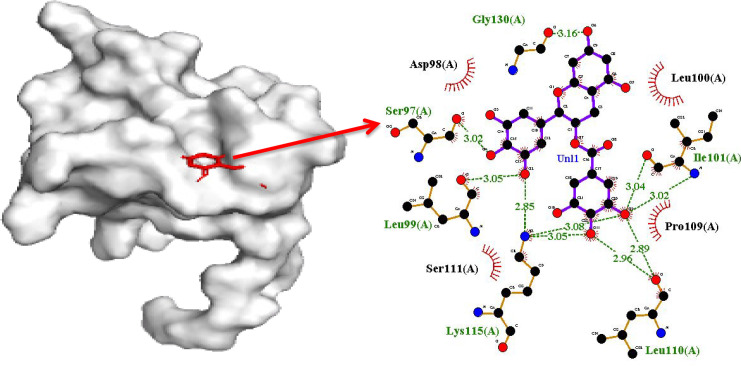
Protein Ligand Complex Representation for c-terminal Zinc Binding Domain of E6 Oncoprotein with Ligand Epigallocatechin Gallate (EGCG)

**Figure 4:**
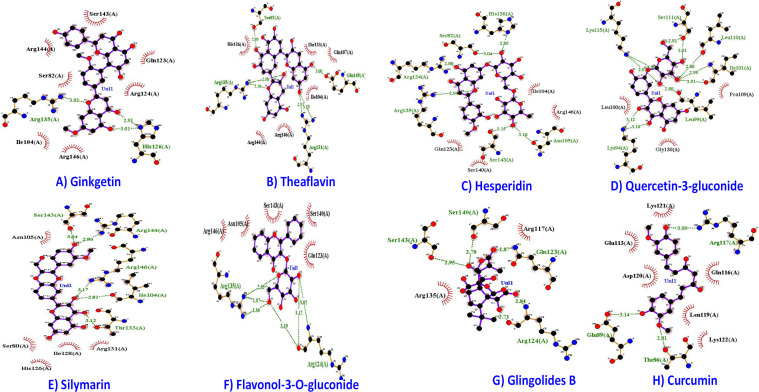
Molecular Docking Complex of natural Compounds with E6 Oncoprotein. A, E6-Ginkgetin; B, E6- Theaflavin; C, E6-Hesperidin; D, E6-Quercrtin-3-gluconide; E, E6-Silymarin; F, E6-Flavonol-3-O-gluconide; G, E6-Glingolides B; H, E6- Curcumin

Another promising compound Epigallocatechin gallate (EGCG), exposed impartial interaction in perspective to 10 H-bonds visualized in Figure 3. The residues interacting with E6 protein in the context to H-bonds are Gly130 (3.16 Å), Ile101 (3.04 Å, 3.02 Å), Leu110 (2.96 Å, 2.89 Å), Lys115 (3.08 Å, 3.05 Å), Leu99 (3.05 Å, 2.85 Å) and Ser97 (3.02 Å). However, residues in hydrophobic connections are Leu100, Pro109, Ser111, and Asp98. These results noticeably establish that EGCG and Withnolide D target the common binding site of oncoprotein. Molecular interaction of E6 oncoprotein with the natural compounds is depicted in Figure 4. Binding energy of Silymarin -6.7 kcal/mol. and the interacting residues of E6 oncoprotein in the context to hydrophobic contact and H-bond are Arg131, Ile128, His126, Ser80,Asn105 and Arg144 (2.86 Å), Arg146 (3.17 Å), Ile104 (2.81 Å), Thr133 (3.12 Å) and Ser143 (3.04 Å) .

**Figure 5:**
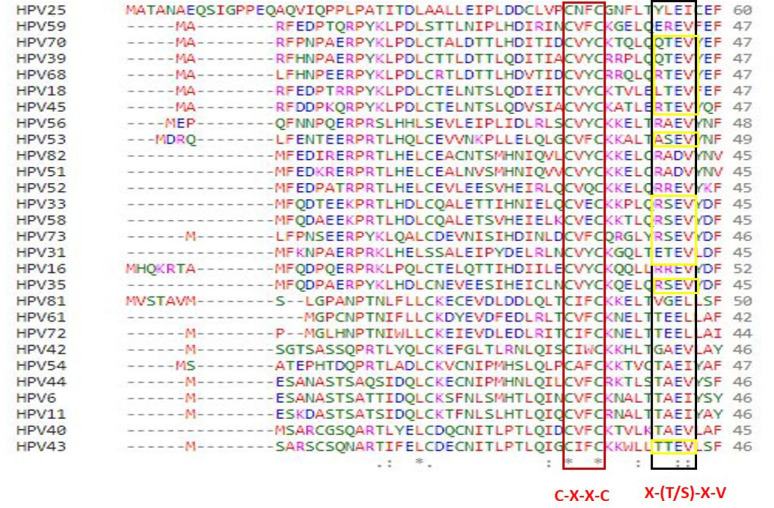
Multiple Sequence Alignment for E6 Oncoprotein of 28 HPVs

Multiple sequence alignment commonly used algorithms for the set of biological sequences (RNA, proteins, DNA). The aim of a MSA method is to align the sequences in a way that will either reflect their evolutionary, functional or structural relationship (Chatzou et al., 2016). In multiple sequence alignment, zinc finger domain and PDZ binding motif were identified in HPV E6 (Figure 5). The CXXC zinc finger domain motif is present in all HPV E6 protein sequences. The E6 protein of HPV consists of 158 amino acid residues and contains (Cys-X-X-Cys) zinc fingers. This zinc finger sequence motif is unique for papillomavirus E6 and E7 proteins and includes precise amino acid residues, extremely conserved among all carcinogenic HPVs as well as many animal and human papillomaviruses associated with benign lesions. The zinc finger domain was identified in all variants of HPV E6 oncoprotein. Zinc finger domain is crucial in DNA recognition, RNA packaging, transcriptional activation, protein folding and assembly (Nedecky et al., 2013).

PDZ domain (X-T/S-X-V) recognition motifs encoded by pathogenic viruses, including high-risk HPVs, target several cellular proteins via protein-protein interactions and those PDZ-containing proteins for proteasome-mediated degradation. In this study high-risk HPV18, HPV31, HPV33, HPV35, HPV39, HPV45, HPV58, HPV68 and HPV73:probable risk HPV53 and low risk HPV43 and HPV70 contains PDZ domain; these HPV-PDZ interactions are in part associated with virally induced cancer progression (Nagasaka et al., 2013).

**Figure 6:**
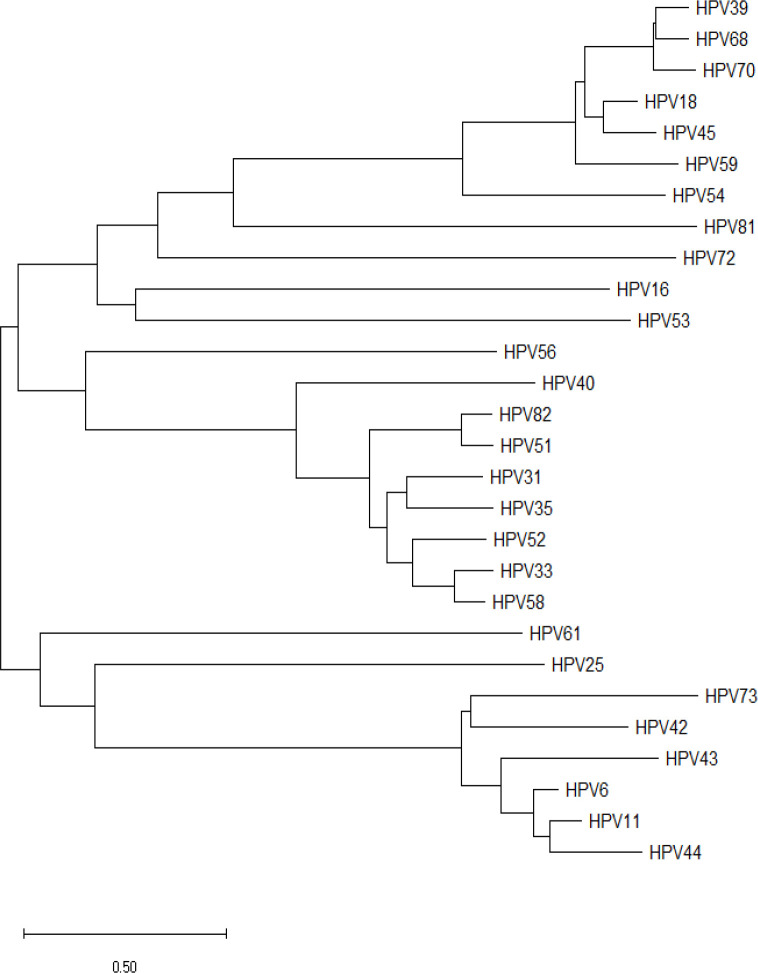
Phylogenetic Analysis for E6 Oncoprotein of 28 HPVs

Phylogenetic analysis offers a detailed understanding of species evolution through genetic changes, analyses the path that joins an organism with its ancestral origin, as well as can predict the genetic variance that may occur in the future. In this study phylogenetic analysis clearly revealed that the high and low-risk strains of HPV E6 proteins have diverse branching (Figure 6). The phylogenetic tree generated using neighbour-joining methods shows that low-risk HPV 42, HPV 43, HPV 44, HPV 6 and HPV 11 are closely related. However, high-risk HPV 73 is an exception. Low risk HPV E6 oncoprotein leads to the degradation of p53, but it is capable of trapping p53 in the cytoplasm. So, the low risks HPVs are not being able to cause cancer. The HPV 25 is evolutionarily close to low-risk HPV61. HPV 56 and 53 are neighbouring to high-risk zone strains especially HPV 16. HPV-16 may probably have a cooperative interaction with HPV-53 in starting neoplastic transformation. It would be likely that HPV-53 maintained the malignant phenotype induced by HPV-16 and subsequently induced the switch of high-grade intraepithelial lesions into invasive cancer (Zappacosta et al., 2014). The low-risk HPV 72 and HPV 81 are exceptional and interrelated to high-risk HPV 16 and HPV 54 subsequently.

Retreatment with platinum-based combination therapy offers some improvement in survival as compared to no therapy. A randomized trial of cisplatin versus the combination of cisplatin and paclitaxel demonstrated improved response rates for the combination but no impact on survival, which remained in the 8–9 month range. Recent trials in this patient population include the addition of targeted therapies such as cetuximab (GOG207), taxanes (GOG240) or immunotherapies (GOG265). HPV-induced progressive diseases are associated in absence of strong HPV-specific CD4+ and CD8+ T cell response and rather, are infested with immunosuppressive cells (Stern et al., 2012). In some circumstances, the balance of positive and negative immune factors may be changed, leading to clearance of lesions. This is an area where therapeutic vaccines are used against HPV.

**Table 1:** Types of HPVs with UniProtKB Accession Identifiers

HPV Type	HPV Group	Accession I’D of UniProtKB
HPV 16	High Risk	P03126
HPV 18	High Risk	P17386
HPV 31	High Risk	P17386
HPV 33	High Risk	P06427
HPV 35	High Risk	P27228
HPV 39	High Risk	P24835
HPV 45	High Risk	P21735
HPV 51	High Risk	A0A0P0EGJ1
HPV 52	High Risk	Q4TUH7
HPV 58	High Risk	Q8QHQ3
HPV 59	High Risk	Q81964
HPV 68	High Risk	P54667
HPV 73	High Risk	Q82005
HPV 82	High Risk	Q9IR59
HPV 25	Probable Risk	P28833
HPV 53	Probable Risk	P36815
HPV 56	Probable Risk	P24836
HPV 6	Low Risk	B6ZCR2
HPV 11	Low Risk	P04019
HPV 40	Low Risk	P36812
HPV 42	Low Risk	P27229
HPV 43	Low Risk	P19709
HPV 44	Low Risk	I7K4T7
HPV 54	Low Risk	Q81018
HPV 61	Low Risk	Q80948
HPV 70	Low Risk	P50804
HPV 72	Low Risk	Q81997
HPV 81	Low Risk	A0A1P8NVU8

**Table 2:** Molecular Docking Results of Natural Compounds with E6 Oncoprotein

Sr. No.	Ligand Name	Binding energy (kcal/mol)	No. of Hydrogen bond	No. of hydrophobic interactions
1	Withanolide D	-7.5	4	6
2	Ginkgetin	-7.3	3	7
3	Theaflavin	-7.2	6	6
4	Hesperidin	-7.1	6	4
5	Quercetin-3-gluconide	-7.1	8	3
6	Silymarin	-6.9	5	5
7	Epigallocatethin gallate (EGCG)	-6.7	10	4
8	Flavonol 3-O-glycoside	-6.7	6	5
9	Ginkgolides B	-6.6	5	2
10	Curcumin	-6.5	3	6

## Discussion

Viruses behaving as oncogenes through a variety of molecular processes end up with neoplasia. HPV oncoprotein, E6, forms complexes with cellular proteins and triggers modulation, such as mitigating telomere shortening, immortalization, host cell differentiation, controlling cellular pathways, regulating growth factors, tumour suppressor degradation and inactivation, disruption with DNA repair efficiency, and apoptosis and facilitate cell transformation, and increment of the hTERT gene (Nabati et al., 2020). HPV is the key factor for developing cervical cancer in women. One of the most serious and lethal malignancies in women is caused by HPV. Although, surgery, radiation treatment, hormone therapy, chemotherapy combination therapies, and immunotherapy are all possibilities for treating early-stage cervical cancer, there is lacuna for effective prognosis thus no effective cure for a persistent HPV infection. One of the therapeutic options for cervical cancer is herbal extracts; numerous researchers have explored the influence of plant metabolites on cancer treatment. Plant derived herbal metabolites may be one of the solutions for targeting drugs for the HPV driven cancers. Various plant-derived substances have conventionally been investigated separately as promising resources against HPV-caused malignancy. The discovery of new inhibitors from herbal remedies against malignancies such cervical cancer might be possible using benefits from advances in computational approach. Computer-aided drug discovery (CADD) is a valuable tool for analysing the binding interactions between a ligand and its target protein, and it has merged as a dependable, cost-effective, time-saving, and fast method for pharmaceutical research (Desai et al., 2021). The existing virtual screening strongly addresses promising top 10 natural compounds Withanolide D, Ginkgetin, Theaflavin, Hesperidin, Quercetin-3-glucoside, Silymarin, Epigallocatechin gallate (EGCG), Flavonol 3-O-glycoside, Ginkgolides B and Curcumin against E6 oncoprotein. Withanolide D is a flavonoid present in Withania somnifera; it’s anticancer potential is also utilized in breast cancer, head and neck cancer, colon cancer, leukaemia, prostate cancer, thyroid cancer and cervical cancer (Samadi, 2015). EGCG is a polyphenol present in Camellia sinensis. It has the potential against various cancers including colorectal cancer. To enhance the apoptosis (Du et al., 2012). Ginkgetin is a type of bioflavonoid present in the leaves of Ginkgo biloba. Several studies have reported ginkgetin as an anti-cancer drug, anti-tumor potential and anti-viral drug (Park et al., 2017). The flavin is a type of bioflavonoids present in Camellia sinensis. It has an ability to induce apoptosis and inhibit angiogenesis (Gao et al., 2016). Hesperidin is a flavanone glycoside found in citrus fruits. It is an economical by-product of citrus production and one of the most essential bioflavonoids in sweet orange and lemon. Hesperidin has the potential to fight against various cancers (Stanisic et al., 2018). Quercetin-3-glucoside is plant flavonoids present in Salicornia herbacea . Flavonol 3-O-glycoside found in Camellia sinensis plants. And it shows anti-inflammatory, anti-cancer and anti-oxidative potential (Rauf et al., 2018). Ginkgolides B is a biologically active terpene lactone present in the Ginkgo biloba plant. Curcumin polyphenol present in the Curcuma longa plant. Curcumin has been described as a potent antioxidant and anti-inflammatory agent. Evidence has also been presented to suggest that Curcumin can suppress tumour initiation, promotion and metastasis (Aggarwal et al., 2003). Quercetin-3-glucoside shows inhibitory effects against various cancers such as breast cancer, head and neck cancer, colon cancer, pancreatic cancer, liver cancer, blood cancer and cervical cancer (Bishayee et al., 2013). Silymarin is a bioflavonoid present in Silybum marianum. Silymarin also has a variety of anti-cancer as well as antiviral properties (Delmas et al., 2020). Few recent in-silico analysis on HPV show the importance of computational approach in drug designing. Curcumin and EGCG also reported against E6 oncoprotein of HPV. Curcumin, Epigallocatechin-3-gallate (EGCG), Jaceosidin, Resveratrol, Indole-3- carbinol, Withaferin A, Artemisinin, Ursolic acid, Ferulic acid, Berberine, Gingerol, and Silymarin are possible effective sources of cancer treatment (Lin et al., 2020). Colchicine, Curcumin, Daphnoretin, Ellipticine, Epigallocatechin-3-gallate have potential against E6 and E7 oncoproteins of HPV 16 and HPV 18 (Mamgain et al., 2015). Withaferin A, Silymarin, Ferulic acid EGCG , Indole-3- carbinol, Artemisinin, Jaceosidin, Resveratrol, Ursolic acid, Berberine, 5 Gingerol - Curcumin are inhibitor against E6 oncoprotein of HPV 18 (Kumar et al., 2015). Artemisinin, Withaferin A, Ursolic acid, Ferulic acid, EGCG, Indole-3-carbinol, Silymarin, Indole-3-carbinol and Gingerol have the capability of inhibiting the E6 oncoprotein of HPV 16 and HPV 18 (Kumar et al., 2016).

The E6 oncoprotein is the prime hotspot region involved in carcinogenesis. The PDZ domain-binding motif appears to be particularly crucial for neoplastic transformation in cultured cells, transformation of primary human keratinocytes, and hyperplasia and carcinogenesis in E6-transgenic mice (Yoshimatsu et al., 2017). C-terminal PDZ-binding motif is particularly conserved among E6 proteins of high-risk HPVs (Thatte et al., 2018). However, the multiple sequence alignment has shown the presence of PDZ-binding motif in all high-risk types along with low-risk HPV 43, HPV70 and probable risk HPV 53. The study of evolutionary links among molecules, traits, and species is known as phylogenetic analysis. The physicochemical characteristics of nucleic acids or amino acids are critical factors that impact their structures or activities, therefore it may give function prediction in the case of infections and pathogenicity. Relationship study of some isolates, as seen in the phylogenetic tree, may yield different results when other genes or proteins are employed as points of reference. The current study reveals the existence of a PDZ binding motif in low-risk HPV 43, HPV 70, and HPV 53, which makes them plausible disease-causing candidates, leading to evolutionary proximity to high-risk HPV. The determination of related phylogenetic categorization or the naming of HPV types is based on one of the most conserved L1 genes (De Villiers et al., 2004). One more study shows the relation of polymorphism in HPV and infection. The phylogenetic tree results for HPV E6 variant proteins revealed a distinctive pattern association, In this study HPV 72, HPV 81, HPV 56 and HPV 53 exhibited inconsistency for which branch it belongs to and revealed that HPV E6 variants have various biological and biochemical consequences (Giannoudis et al., 2001), which have changed carcinogenic potential. Computational characterization of human papillomavirus variations plays a crucial role in the HPV driven cancer prognosis, development of vaccines, and other treatment approaches to address virus-induced disorders. The present study limits the pharmacogenomics approach to determine the specific mechanism and downstream pathways.

In conclusion, within the limitations of the present study, Withnolide D, Ginkgetin, Theaflavin, Hesperidin, Quercetin-3-glucoside, Silymarin, Epigallocatechin gallate (EGCG), Flavonol 3-O-glycoside, Ginkgolides B and Curcumin were identified as potential inhibitors of HPV 16 E6 protein. The inhibitory effect of natural compounds against E6 protein has been studied , thus, advancements in computational science and bioinformatics are beneficial for the study of novel inhibitors from natural sources. The E6 protein of HPV 16 inactivates p53; therefore, the process of gene regulation is disturbed, which is a fundamental cause of cervical cancer. Thus, E6 protein of HPV-16 is of considerable interest for discovery and designing of novel molecules to overcome the challenges. This computational analysis reveals that it is a promising platform for developing anti-cancerous competitive inhibitors targeting HPV. In-silico analysis of E6 protein reveals that all HPV E6 proteins contain PDZ domain and zinc finger domain. The phylogenetic analysis demonstrates that HPV E6 low risk protein and high risk protein are distinct from each other except HPV 73, HPV 72 and HPV 81.In future, this computational characterization will have significant impact on wet-lab studies.

## Author Contribution Statement

The conception and design of study by SS & MP,acquisition of data, analysis and manuscript drafting by TJ, interpretation of data by TJ & MP and manuscript editing by SS.
